# Polymer Networks for Enrichment of Calcium Ions

**DOI:** 10.3390/polym13203506

**Published:** 2021-10-12

**Authors:** Marcus Heinze, Christoph Horn, Doris Pospiech, Regine Boldt, Oliver Kobsch, Kathrin Eckstein, Dieter Jehnichen, Brigitte Voit, Stefan Baudis, Robert Liska, Anna Naumova, Kay Saalwächter, Urs Lendenmann, Norbert Moszner

**Affiliations:** 1 Leibniz-Institut für Polymerforschung Dresden e.V., Hohe Str. 6, 01069 Dresden, Germany; marcus.heinze@better-basics.de (M.H.); horn-christoph@ipfdd.de (C.H.); boldt@ipfdd.de (R.B.); kobsch@ipfdd.de (O.K.); eckstein@ipfdd.de (K.E.); djeh@ipfdd.de (D.J.); voit@ipfdd.de (B.V.); 2 Organic Chemistry of Polymers, Technische Universität Dresden, 01069 Dresden, Germany; 3 Better Basics Laborbedarf GmbH, Zwickauer Str. 69, 01159 Dresden, Germany; 4 Institut für Angewandte Synthesechemie, Technische Universität Wien, Getreidemarkt 9/163, 1060 Wien, Austria; stefan.baudis@tuwien.ac.at (S.B.); robert.liska@tuwien.ac.at (R.L.); 5 NMR, Institut für Physik, Martin-Luther Universität Halle-Wittenberg, Betty-Heimann-Str. 7, 06120 Halle, Germany; anna.naumova@physik.uni-halle.de (A.N.); kay.saalwaechter@physik.uni-halle.de (K.S.); 6 Ivoclar Vivadent AG, Bendererstr. 2, FL-9494 Schaan, Liechtenstein; Urs.Lendenmann@ivoclarvivadent.com (U.L.); norbert.moszner@ivoclarvivadent.com (N.M.)

**Keywords:** solvogel, hydrogel, polymer buffer, phosphonic acid, network morphology, dental materials

## Abstract

In this study, solvogels containing (2-((2-(ethoxycarbonyl)prop-2-en-1-yl)oxy)-ethyl) phosphonic acid (ECPA) and *N*,*N*′-diethyl-1,3-bis-(acrylamido)propane (BNEAA) as the crosslinker are synthesized by UV induced crosslinking photopolymerization in various solvents. The polymerization of the ECPA monomer is monitored by the conversion of double bonds with *in situ* attenuated total reflection Fourier transform infrared (ATR-FTIR) spectroscopy. The morphology of the networks is characterized by *in situ* photorheology, solid state NMR spectroscopy, and scanning electron microscopy (SEM) of the dried gels. It is demonstrated that the storage modulus is not only determined by the crosslinker content in the gel, but also by the solvent used for preparation. The networks turn out to be porous structures with *G′* being governed by a rigid, phase-separated polymer phase rather than by entropic elasticity. The external and internal *pK_a_* values of the poly(ECPA-*co*-BNEAA) gels were determined by titration with a specially designed method and compared to the calculated values. The polymer-immobilized phosphonic acid groups in the hydrogels induce buffering behavior into the system without using a dissolved buffer. The calcium accumulation in the gels is studied by means of a double diffusion cell filled with calcium ion-containing solutions. The successful accumulation of hydroxyapatite within the gels is shown by a combination of SEM, energy-dispersive X-ray spectroscopy (EDX) and wide-angle X-ray scattering (WAXS).

## 1. Introduction

Hydrogels continuously attract high interest both scientifically and application-wise since they contain large amounts of water or biological fluids. Hydrogels are networks consisting of synthetic or natural polymer chain segments crosslinked either physically or chemically. The polymer network is typically swollen by water, which contributes to the biocompatibility of various hydrogels [1,2,3,4]. For this reason, hydrogels represent important materials in medicine and are used in various biomedical fields, e.g., as substrates in tissue engineering, as materials for contact lenses, as containers for drug delivery, for wound healing materials, and for cell encapsulation [5,6,7,8]. Hydrogels have also found special applications in dentistry (for instance, in self-etching adhesives and restorative dentistry) [9,10,11,12,13]. These applications often call for additional functions, such as the ability to adhere on dentin substrates, which can be provided by tailor-made monomers with special chemical structures. These structures often bear phosphonic or phosphoric acid groups [14,15,16,17,18]. Their incorporation in hydrogels requires a certain hydrolytic stability of the linkage between phosphonic acid and polymer backbone [19,20].

All applications of hydrogels rely on well-understood structure-morphology-property relationships [21]. In this contribution, multifunctional gels consisting of monomers presently already used in dental networks [19] were designed, prepared, and studied. The networks were intended to generate hydrogels in water, which are able to stabilize the pH value in a certain environment (i.e., buffering). This internal buffering effect could protect teeth from acid attacks. To achieve this, a buffering region above *pK_a_* 5.5 has to be reached [22]. Moreover, the presence of phosphonic acid groups within the hydrogels should enhance the enrichment of calcium ions from saliva. These calcium ions together with phosphate ions can precipitate and form hydroxyapatite which is known as important constituent of teeth and bones. Thus, a remineralization in the environment of the phosphonic-acid-containing hydrogels was expected to occur. Dalas et al. [23] reported the precipitation of hydroxyapatite on the powder of polyamide with diethoxyphosphinyl substituents. We were interested to find out whether or not such a process would also occur in the hydrogel systems studied.

Phosphonic-acid group containing monomers were incorporated into the hydrogels studied here. Such monomers find also applications in self-etching adhesives due to their high attraction to calcium contained in dental hydroxyapatite. One of these monomers is (2-((2-(ethoxycarbonyl) allyl)oxy)ethyl)phosphonic acid (ECPA), which shows improved hydrolytic stability under acidic conditions since the phosphonic acid is linked to the methacrylate via an ether bridge [9,14]. The copolymerization behavior of this monomer with HEMA as well as itaconic acid has been reported recently [24,25].

**Figure 1:**
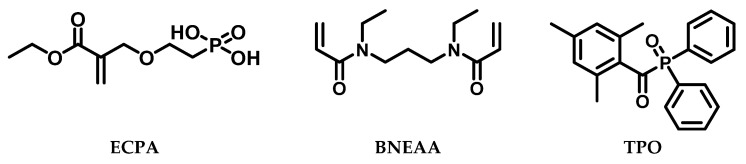
Chemical structures of phosphonic monomer ECPA, crosslinker BNEAA, and photo initiator TPO used in this study.

The networks in this study were prepared by UV irradiation-induced crosslinking photopolymerization by using the photoinitiator (2,4,6-trimethylbenzoyl)diphenylphosphine oxide (TPO). The chemical structures of the compounds used are shown in Figure 1. The phosphonic acid functionalities of the monomer ECPA were intended to generate a buffering effect in the hydrogels to adjust the pH in the range between 5.5 to 6.4. As crosslinker (CL), *N*,*N*′-(propane-1,3-diyl)bis(*N*-ethyl acrylamide) (BNEAA) was employed, a compound used in dental formulations [18,26]. Solvents with stepwise varied polarity (dimethyl sulfoxide, ethanol, iPrOH, propylene glycol, water, and mixtures of ethanol with water) were employed for the *in situ* generation of swollen polymer networks (solvogels).

The influences of molar composition and solvent on the formation of the solvogels, the gel morphology and the properties of the networks were studied with a combination of suitable methods. *in situ* attenuated total reflection Fourier transform infrared (ATR-FTIR) spectroscopy was used to analyze the polymerization of phosphonic acid monomer, while photorheology was employed to follow the crosslinking polymerization of ECPA with the crosslinker BNEAA. The plateau moduli *G′* were monitored to follow the macroscopic network formation in real time. The morphology of the water-swollen networks was analyzed by solid state NMR spectroscopy, amended by swelling measurements. The heterogenous microstructure of freeze-dried gels was imaged by scanning electron microscopy (SEM). All results were combined to derive a structural model for the phosphonic acid groups-containing hydrogels in dependence on the solvent used for preparation as well as the CL content.

Phosphonic acid groups chemically linked in a hydrogel network are assumed to induce buffering behavior into the hydrogel without using a buffer solution. Therefore, the acid-base behavior was analyzed by a special titration method developed to adapt to the slower diffusion of ions in swollen networks. Furthermore, SEM with energy-dispersive X-ray spectroscopy (EDX) and wide-angle X-ray scattering (WAXS) were used to study the calcium ion adsorption of the water-swollen networks treated with calcium-containing liquids. These measurements should allow conclusions about the hydroxyapatite mineralization ability of the hydrogel.

## 2. Materials and Methods

### 2.1. Materials

Calcium hydroxide (98%, ACROS Organics B.V.B.A, Fair Lawn, NJ, USA), calcium nitrate tetrahydrate (Ca(NO_3_)_2_ × 4 H_2_O, 99%, Merck KGaA, Darmstadt, Germany), deionized water (Millipore^®^, Merck KGaA, Darmstadt, Germany), dimethyl sulfoxide (>99.5%, Sigma-Aldrich Chemie GmbH, Munich, Germany), (2-((2-(ethoxycarbonyl) allyl)oxy)ethyl)phosphonic acid (ECPA, Ivoclar Vivadent AG Schaan, Liechtenstein), ethanol (99.9%, VWR International GmbH, Radnor, PA, USA), hydrochloric acid, (HCl, 0.1 mol/L Titrisol^®^, Merck KGaA, Darmstadt, Germany), isopropanol (analytical grade, Thermo Fisher Scientific GmbH, Dreieich, Germany), *N*,*N*′-(propane-1,3-diyl)bis(*N*-ethylacrylamide) (Ivoclar Vivadent AG Schaan, Liechtenstein), potassium phosphate tribasic (K_3_PO_4_, ≥98%, Sigma-Aldrich Chemie GmbH, Munich, Germany), propylene glycol (99%, ACROS Organics B.V.B.A, Fair Lawn, NJ, USA), sodium hydroxide (NaOH, 0.1 mol/L Titrisol^®^, Merck KGaA, Darmstadt, Germany) (2,4,6-trimethylbenzoyl)diphenylphosphine oxide (97%, Sigma-Aldrich Chemie GmbH, Munich, Germany) were used as received.

### 2.2. Preparation of In Situ Swollen Free-Standing Solvogels

Solvogels with varied composition as summarized in Table S1 containing the phosphonic acid monomer ECPA and the crosslinker BNEAA were synthesized by UV irradiation-induced crosslinking photopolymerization with TPO as photoinitiator. The solvent content of all gels was 80 wt.% based on the total mass of the system. Water, ethanol/water (50/50 vol.%/vol.%) (EtOH/H_2_O (50/50)), ethanol/water (80/20 vol.%/vol.%) (EtOH/H_2_O (80/20)), propylene glycol (PG), isopropanol (iPrOH) and dimethyl sulfoxide (DMSO) were used as solvents. ECPA, BNEAA, TPO were dissolved and mixed in the corresponding solvent to a homogeneous solution in a light-protected glass vessel. Example for the sample poly(ECPA-*co*-BNEAA) (19/1): ECPA (0.95 g, 3.989 mmol), BNEAA (0.05 g; 0.210 mmol), TPO (0.025 g; 0.072 mmol) and solvent (4 g) were mixed. Prior to polymerization, the reaction mixture was degassed by nitrogen flow and placed between two glass plates sealed with a rubber spacer (thickness 2 mm). The UV-induced polymerization was carried out for 10 min on each site at a wavelength of 365 nm with a Herolab 8W UV analysis lamp (Herolab GmbH, Wiesloch, Germany) fixed horizontally at a distance of 10 cm from the center of the syringe. After irradiation, the mold was opened and the gel film was immersed in deionized water for one week to remove non-reacted monomers, changing the water once a day. If necessary, the solvogels were freeze-dried to generate xerogels.

### 2.3. Methods

#### 2.3.1. Quantitative Analysis of the Soluble Contents in the Gel Samples

The mold used to prepare the gel films was weighed before and after filling with the monomer/solvent mixture. The theoretical polymer content was calculated from the formulation. Then, the monomer/solvent mixture was UV polymerized and dialyzed with water as described before. The water portions used for dialysis were collected and the water was removed by evaporation, giving the content of soluble material in the gel samples. The dialyzed gels were freeze-dried and weighted, giving the crosslinked amount of the sample.

#### 2.3.2. *In Situ* ATR-FTIR Spectroscopy

*In-situ* attenuated total reflection-Fourier-transform infrared (ATR-FTIR) spectroscopy was used to obtain the time-dependent double bond conversion during UV homopolymerization of ECPA. A commercial ATR-FTIR attachment operated by the single-beam-sample-reference (SBSR) concept (OPTISPEC, Zürich, Switzerland) was used [27]. The ATR-FTIR attachment was installed on a Tensor 27 FTIR spectrometer (Bruker Optics, Ettlingen, Germany) equipped with a globe source and a mercury cadmium telluride (MCT) detector. For transmission (TRANS) FTIR experiments, a deuterated triglycine sulfate (DTGS) detector was used and 100 scans at a spectral resolution of 2 cm^−1^ were accumulated for TRANS spectra. The intensity of the sample (*I_S_*) and the reference (*I_R_*) were measured to calculate the ATR absorbance (*A*) by the Beer-Lambert law *A* = −log(*I_S_*/*I_R_*).

The monomer mixtures were prepared as described above, but without CL BNEAA. 20 wt.% ECPA in 80 wt.% ethanol/water mixture (50/50 vol.%/vol.%) with 0.5 wt.% TPO as initiator was used. The monomer solution was injected into the *in-situ* ATR-FTIR measuring cell using a syringe and the measuring cell was then inserted into the spectrometer. This cell consisted of two partially UV-transparent plates sealing the upper and lower halves of the front and back side of a germanium internal reflection element (IRE, 50 × 20 × 2 mm^3^) with O-rings. After rinsing the respective compartment, the set-up was flushed with nitrogen and the reference spectrum was recorded. Then, the measuring cell was removed and irradiated vertically at a wavelength of 365 nm with a Herolab 8 W UV analysis lamp (Herolab GmbH, Wiesloch, Germany) at a distance of 10 cm for the respective reaction time (1 to 15 min). The measuring cell was then reinserted into the spectrometer and after rinsing the set-up again with nitrogen, the next spectrum was recorded.

#### 2.3.3. Photorheology

Photorheological measurements according to [28] were carried out using a rheometer MCR302 WESP (Anton Paar GmbH, Graz, Austria) in plate/plate geometry with a diameter of 25 mm at a temperature of 25 °C with a sample volume of 70 µL, a slit size of 100 µm, and a frequency of 1 Hz. The procedure and setup has already been reported in detail in ref. [28]. The UV light source was an OmniCure 2000 (Exfo, Mississauga, ON, Canada) with a wave length of 320–500 nm and a light power of 30 mW·cm^−2^, calibrated at the sample position with a spectrometer USB 2000+ (Ocean Optics, Winter Park, FL, USA). A transient phase of 60 s and an irradiation time of 300 s were applied. The measurements were repeated three times and averaged. From the plateau moduli *G’*, the following network parameters were calculated according to [29] for samples for which the validity of the classical rubber-elasticity theory behavior and the validity of the Flory theory can be assumed [30,31]:

Effective crosslink density *ν_eff_* according to the phantom model [29]:

(1) $$\nu_{eff}=\frac{G\prime }{ART\eta }$$

(*G′*, plateau value of the storage modulus; *A*, microstructure factor (with *A* = 1 − (2/*f*), *f* being the crosslink functionality, set to 4); *R*, gas constant; *T*, temperature; *η*, memory term, set to 1).

Chemically expected crosslink density *ν_chem_*:

(2) $$\nu_{chem}=\frac{{fn}_{crosslinker}}{2V}$$

(*f*, functionality; *n_crosslinker_*, molar concentration of CL; *V*, volume).

Number-average molecular weight of the chain between two junction points *M_c_* [29]:

(3) $$M_{c}=\frac{\rho_{Polymer}}{\nu }$$

(*ρ*_Polymer_, density of the polymer).

#### 2.3.4. Solid State NMR Spectroscopy

The samples for ^1^H solid-state NMR measurements were prepared with different ratios of ECPA/BNEAA ranging from 10/10 wt.% to 19.5/0.5 wt.%, and constant content of TPO (0.5 wt.% with respect to the complete sample) in the solvent PG. After irradiation (20 min at 365 nm, 8 W, distance of 10 cm), the samples were dialyzed against deuterated water (D_2_O), exchanging the solvent at least eight times. The NMR measurements were performed in the D_2_O-swollen state in 10 mm sample tubes filled to about 8 mm height to restrict the samples to the region of homogeneous B_0_ field. A Bruker Minispec mq20 (Bruker, Karlsruhe, Germany) with a ^1^H Larmor frequency of 19.9 MHz (*B*_0_ ~ 0.5 T) was used, providing 90° pulses of about 2.3 µs length and featuring a dead time of about 12 µs.

Simple free-induction decays (FIDs) were measured to quantify polymer components with different overall mobility as distinguished by different transverse relaxation times (*T*_2_). Magic sandwich echo (MSE) was used to overcome the instrumental dead time before detection which suppresses immobile-component signals, in particular due to imperfections. Therefore, fits were performed on data detected after both a single 90° pulse (which provides quantitative signal amplitude but is subject to the dead-time problem) and MSE refocusing. A joint fit then enables to obtain reliable component amplitudes following procedures previously published [32]. To better characterize polymeric components acting as entropically active network chains (featuring a finite residual dipolar coupling constant *D_res_* arising from the end-fixation of otherwise freely fluctuating chains), multiple-quantum (MQ) NMR using an improved pulse sequence based on the experiment of Baum and Pines [33] was applied, as discussed in detail in previous work [34,35].

#### 2.3.5. Scanning Electron Microscopy (SEM)

The dialyzed as well as mineralized hydrogels were freeze-dried, cut perpendicularly to the surface, and the morphology of the internal porous structure was imaged by SEM Ultra Plus (Carl Zeiss AG, Oberkochen, Germany) equipped with SE2 detector. The measuring voltage was 3 kV, the aperture size 30 µm, and as scan method line average was chosen. SEM was able to detect pores larger than 10 nm. The averaged pore size was determined visually by averaging the diameter of the pores in the SEM images (values averaged from 10 positions).

#### 2.3.6. Titration of Swollen Networks

Acid-base titration with incremental titrant addition of 0.1 mL⋅180 s^−1^ was performed by means of a Mettler Toledo titrator T70 (Mettler Toledo, Columbus, OH, USA) equipped with pH electrode (Mettler Toledo DG111-SC). NaOH (0.04 mol/L) or Ca(OH)_2_ (0.02 mol/L) and HCl (0.04 mol/L) were used as titrants.

A modified density determination set for analytical balances was used to weigh swollen gel samples for titration. A fine-pored sieve pan was suspended from a balance and completely immersed in water. After taring the balance, the swollen gel was placed on the sieve pan in a way that the gel was completely immersed in water and was only in contact with the sieve pan. For each gel composition, three gel samples of different sizes were weighed first under water and then after complete drying in air. The ratios obtained from this procedure were used to weigh swollen gel films of corresponding composition under water.

#### 2.3.7. Energy-Dispersive X-ray Spectroscopy (EDX)

The freeze-dried poly(ECPA-*co*-BNEAA) gels were fixed to aluminum pins with a double-sided adhesive carbon tap. To avoid charging, the samples were covered with a thin film of carbon by evaporating a carbon fibre at 10^−4^ mbar with a BalTec SCD500 sputter coater (Leica Microsystems GmbH, Wetzlar, Germany).

EDX measurements were performed using a SEM Ultra Plus (Carl Zeiss AG, Oberkochen, Germany) equipped with an EDX detector XFlash 5060F (Bruker Nano GmbH, Berlin, Germany). An accelerating voltage of 6 keV was used for the analysis of light elements, and a voltage of 12 keV was used for the analysis of elements with higher atomic number. The samples were examined at different positions with various magnifications.

#### 2.3.8. Wide-Angle X-ray Scattering (WAXS)

The freeze-dried gels were characterized by WAXS performed with the 2-circle diffractometer XRD 3003 T/T (GE Sensing & Inspection Technologies, Ahrensburg, Germany) in symmetric step-scan mode with Δ2*θ* = 2° in the range of 2*θ* = 5–45° and *t* = 40 s (using 40 kV/30 mA CuKα radiation with *λ* = 0.1542 nm, monochromatized by primary multilayer system and slit geometry 0.2 mm/0.3 mm). The scattering results were presented as *I* versus 2*θ*. All diffractograms were normalized and compared to reflections of hydroxyapatite.

## 3. Results and Discussion

**Scheme 1:**
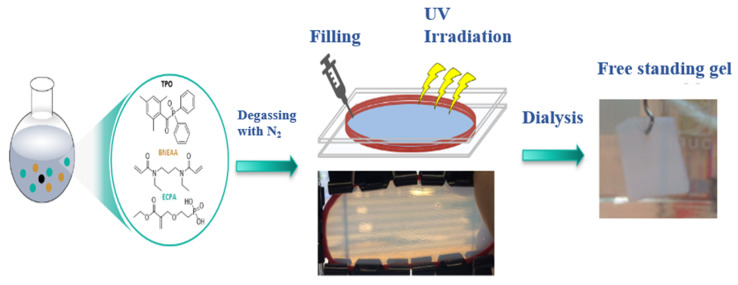
Schematic illustration of the preparation of poly(ECPA-*co*-BNEAA) solvogels.

The solvogel polymer networks were prepared as described in the Experimental part *in situ* in the respective solvent or solvent mixture. The preparation is illustrated in Scheme 1. The resulting films were dialyzed with deionized water after photopolymerization to remove non-reacted monomers, solvent and non-crosslinked polymers, and were characterized as reported in the following. The composition of all solvogels prepared is summarized in Table S1.

### 3.1. Influence of Reaction Conditions on the Solvogel Composition

**Figure 2:**
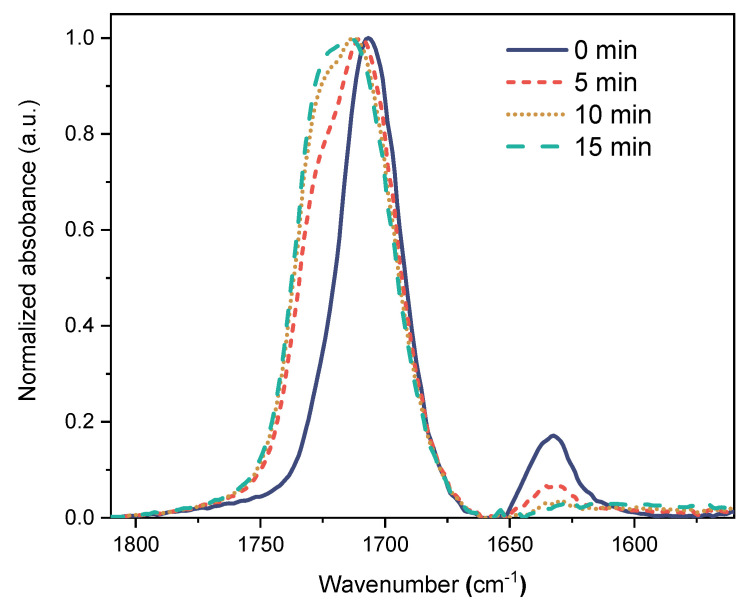
ATR-FTIR spectra taken during the polymerization of ECPA (20 wt.%) in EtOH/H_2_O (50/50) with 0.5 wt.% TPO as initiator after different UV exposure times.

To study the influence of chemical composition and preparation conditions on the network morphology and structure, and finally their properties, it was necessary to follow the conversion of monomers during the gel formation in the polymerization system (20 wt.% monomer + CL, in the appropriate solvent, with 0.5 wt.% photoinitiator). Both, ^1^H NMR and Raman spectroscopy did not provide clear results. The forming gel did not give high resolution NMR signals due to the restricted segmental mobility. The Raman bands of C=C double bonds detected in the reaction mixture were too weak to give information. Therefore, *in situ* ATR-FTIR spectroscopy was employed using a special measuring cell (see Supporting Material (SM), Figure S1). The main aim was to find optimum reaction conditions for high monomer conversions. The measurements were carried out for the homopolymerization of ECPA without CL. When CL was incorporated, the forming gels sticked to the ATR window and did not allow further monitoring of the proceeding reaction. However, the results with ECPA were useful to understand the formation of crosslinked gels as well. Figure 2 illustrates the course of photopolymerization of ECPA (20 wt.% in ethanol/water mixture 50/50 vol.%/vol.% with 0.5 wt.% TPO as photoinitiator) after different UV exposure times. The signal of the C=C double bond at 1635 cm^−1^ decreased over time, and the range between 1610 and 1660 cm^−1^ was used to calculate the degree of conversion in different solvents, as summarized in Table 1.

Double bond conversions > 80% were found after 15 min UV exposure in polar solvents (DMSO, EtOH/H_2_O, iPrOH), while the conversion in the less polar solvent PG was low at about 22%. The molar monomer concentration did not influence the result significantly (Table 1).

All ECPA double bond conversions were found to be below 100% (see Table 1). Increasing the reaction time beyond 15 min exposure did not raise the conversion further. Therefore, the networks obtained by copolymerization with the CL BNEAA were dialyzed before analysis to remove residual monomers. The dialysate was concentrated and analyzed by ^1^H NMR spectroscopy. Residual monomer (ECPA) and homopolymer (poly(ECPA)) were only found after polymerization in iPrOH, as indicated in Table 2. This means that the molar composition of the network corresponded to the composition of the educts (except for polymerization in iPrOH) under the polymerization conditions used.

### 3.2. Determination of the Crosslinker Content Necessary to Form Macroscopic Gels

The results discussed above indicated the strong influence of the solvent on the polymerization of ECPA. An UV irradiation time of least 15 min was required to achieve a suitable monomer conversion. A further influence on the network formation is exerted by the CL. Therefore, the content of CL within the total monomer amount of 20 wt.% in different solvents using 0.5 wt.% TPO was varied to find suitable conditions for the formation of macroscopic polymer gels. The monomer/CL solutions were irradiated for 60 s with a Bluephase^®^ C8 (mode high). If gelation could not be detected after that time, the irradiation was continued in steps of 30 s up to a maximum of 300 s. The results are summarized in Table S2 (see SM). Solvents with higher polarity required a higher concentration of CL. The necessary CL concentrations in the different solvents rise in the following direction:

PG (0.5 wt.% BNEAA) < DMSO (2.0 wt.% BNEAA) < EtOH/H_2_O (50/50) (3 wt.% BNEAA)

CL contents of more than 3 wt.% generally resulted in the formation of macroscopic polymer gels. Their rigidity depended on the solvent.

### 3.3. Photorheological Monitoring of Network Formation

**Figure 3:**
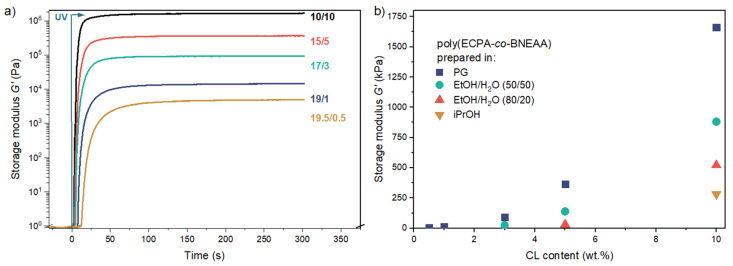
(**a**) Photorheological monitoring of the polymer gel formation in PG using different monomer (ECPA)/crosslinker (BNEAA) ratios (in wt.%/wt.%) with a total amount of 20 wt.% (ECPA + BNEAA = 20 wt.%); (**b**) Storage modulus *G′* at the end of the reaction of poly(ECPA-*co*-BNEAA) networks prepared in different solvents in relation to CL content.

Photorheology has been demonstrated as useful tool to monitor the formation of hydrogels and gels by photopolymerization [28]. The method was applied here for polymer gel formations with different monomer/CL ratios and in different solvents. The storage modulus *G′* in dependence on time was recorded. The results for the gel formation of different ECPA/BNEAA ratios in PG as solvent are displayed in Figure 3. The complete results are summarized in Figure S2 (see SM).

The gelation time was found by fitting the slope of the storage modulus *G′* vs. *t* curves in the linear region and determining the intercept of the fit with the base curve [28]. The results of the ECPA/BNEAA formulations are summarized in Table 3.

The gelation time typically decreased with increasing CL content, and the reaction proceeded faster in more polar solvents such as PG. Precipitation was observed in gels from iPrOH with CL contents lower than 10 wt.%. The plateau values of *G′* obtained from *G′* vs. *t* curves are given in Table S3 (see SM). The values correlate with the mechanical strength of the gels.

The final value of *G′* was found to be the highest in gels obtained from PG, followed by those from EtOH/H_2_O (50/50), EtOH/H_2_O (80/20) and iPrOH. The maximum *G’* rose with increasing amount of CL (Figure 3a), as it could be expected.

The final value of the modulus *G′* could now be interpreted in terms of rubber elasticity theory. However, the discussion of the solid-state NMR results presented in the next section will show that this would be inadequate, as the elasticity of the gels is mostly mediated by particle-particle contacts. Only at the lowest CL contents entropically elastic polymer network chains could be detected. The PG-based gel containing 0.5 wt.% CL was used as an example, for which the phantom model (Equations (1) and (3)) provides a network chain molecular weight *M_c,eff_* of about 370 kg/mol. This can be compared with the chemically expected value *M_c,chem_* (assuming all monomers are part of elastically active chains linked by four-functional linkers) using Equations (2) and (3), which is merely 32 kg/mol. This indicates that this sample must be highly inhomogeneous, with only a fraction of the polymer contributing to the actual elasticity. It is thus of high importance to shed a view on the microstructure of the gels.

### 3.4. Characterization of the Polymer Network Microstructure by Solid State NMR

**Figure 4:**
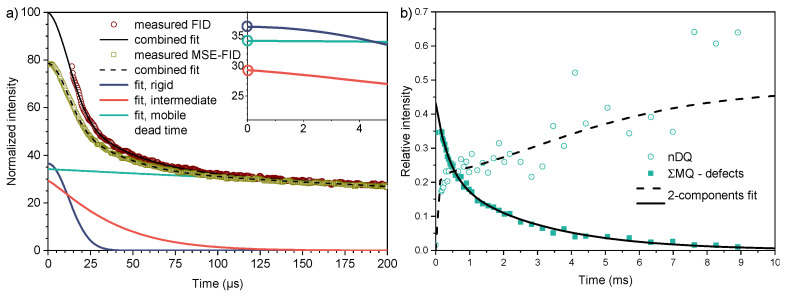
Solid-state NMR results for the poly(ECPA-*co*-BNEAA) samples prepared in PG. (**a**) ^1^H FID NMR measurements of the sample with 3 wt.% CL content and decomposition into components with different mobility. The inset shows the initial time range of the individual fit components, with their percentages marked as y-intercept. (**b**) ^1^H MQ NMR analysis of the constrained fractions in the sample with 0.5 wt.% CL, revealing ca. 17 wt.% network-like chains.

Solid-state ^1^H NMR relaxation experiments are able to differentiate between structural elements with varying mobility [32]. The transverse relaxation behavior of proton signals in swollen networks reflects the molecular dynamics and can be used to distinguish the different components. Figure 4a shows representative FID and MSE-FID results for the PG-based samples with 3 wt.% CL. As a reminder, both reflect the intensity decay due to transverse relaxation, which is rather fast for a part of the sample, indicating the presence of strongly mobility-restricted regions (dissolved linear chains would have a long *T*_2_ and almost no appreciable decay in the studied time range). For a reliable fit of the most constrained part of the sample, the MSE was applied to refocus the signal and start detection at the top of the echo (i.e., at zero time). The MSE was not perfect, showing some loss of signal, but three components with well-distinguished *T*_2_ could be fitted using a combination of three stretched/compressed exponentials $\sim f_{i}\exp{\left\{-t/T_{2,i}\right\}}^{\beta_{i}}$. With the parameters *T*_2*,i*_ and *β_i_* taken from the fit to the MSE, a fit to the FID provided reliable component fractions *f_i_* [32]. The results of this fitting procedure are shown in Figure 4b.

**Figure 5:**
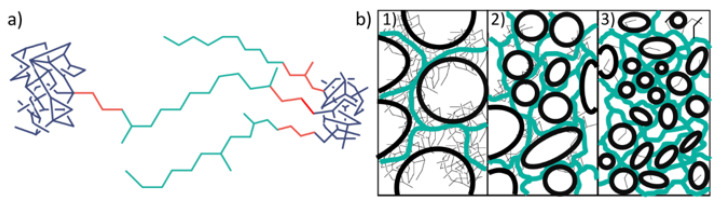
Schematic illustration of: (**a**) rigid (blue), intermediate (red) and mobile (green) segments in polymer networks; (**b**) gels with high content of rigid phases as (**1**) particle gel, (**2**) gel with mixed state and (**3**) high content of mobile phase (swollen elastomer); the green lines represent the elastomeric network, the areas marked black indicate pores.

The mobile component with the longest *T*_2_ always features an exponent *β_m =_*_1_ (i.e., an exponential decay). There are then two constrained fractions, an intermediate one with *T*_2*,int*_ in the order of 30–100 µs and *β_int_* slightly above unity, and a most constrained one associated with quasi rigid material with *T*_2*,rig*_ = 15–20 µs and *β_rig_* = 2. The latter corresponds to the Gaussian decay expected from a rigid (motionless) organic solid, arising from strong dipole-dipole couplings between the protons in the order of 20–30 kHz. Molecular motions on the 20 µs timescale and faster lead to motional averaging and thus reduction of dipolar couplings, explaining the longer *T*_2*,int*_ and *T*_2*,mob*_. Thus, the highly crosslinked networks studied here consist of collapsed, possibly glassy polymer material, mobile dissolved components and an interphase, as illustrated in Figure 5a.

To address the question whether the dissolved chain with the longest *T*_2*,mob*_ in the ms range may be related to elastically active network chains, a more quantitative analysis was required. Physically, network chains (as opposed to defects such as free or dangling chains) feature a finite residual dipolar coupling with coupling constants *D_res_*/2*π* in the 100 Hz range [34,35]. The observed *T*_2*,mob*_ would be compatible with this, but to distinguish between incoherent relaxation effects and signal decay due to a finite *D_res_*, MQ NMR was used to quantify dipolar couplings. In all brevity, this experiment generated two signal functions, a double-quantum (DQ) intensity build-up whose initial rise directly reflects the dipolar coupling constant, and a reference intensity decay curve. The sum of the intensity curves *I*_ΣMQ_ = *I*_DQ_ + *I*_ref_ is a “relaxation-only” function used for normalization purposes. It has a long-*T*_2_ tail associated with network defects, which is already subtracted in the plot of Figure 4b. Those data were taken on the lowest crosslinked sample, which features > 50% defects. The rest of the sample consist of a strongly dipolar-coupled fraction with couplings in the kHz range, corresponding to the intermediate (i.e., partially collapsed) phase from the FID analysis, and an elastomeric component with *D_res_*/2π of order 50 Hz. The latter two were quantified by two-component fits of the two signal functions, using the fitting approach to the normalized DQ intensity *I*_nDQ_ presented in ref. [35] to account for a distribution of couplings, assumed to be log-normal (with moderate width).

**Figure 6:**
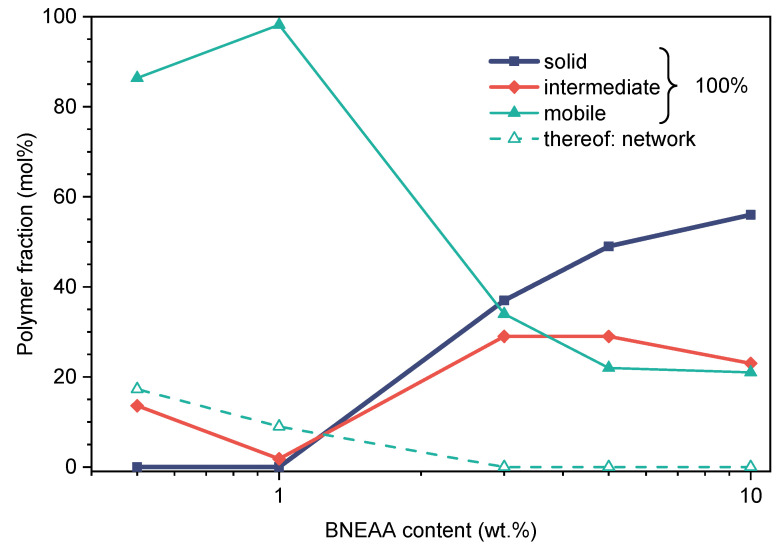
Solid State NMR-based contents of rigid, intermediate and mobile phases as well as network fraction in poly(ECPA-*co*-BNEAA) gels with varying CL content prepared in PG (dark blue, rigid; red, intermediate; green, mobile phase; open/dashed, entropically elastic fraction).

The results of Figure 4b confirm our assumptions on the heterogeneous character of the least crosslinked sample already taken from its low *G′* modulus value. Notably, only the two lowest-crosslinked samples exhibit detectable DQ build-up on the ms timescale, i.e., corresponding to coupling values characterizing network chains. In all other samples, the DQ build-up is restricted to the first, very quick initial rise associated with the collapsed components, which cannot be resolved by the used MQ pulse sequence, but is well quantified by the *T*_2_ values from FID analysis. Figure 6 shows the NMR-detected components of the polymer networks prepared in PG as a function of CL contents. It could be observed that the network-like fraction at low crosslinking was always a minority component, and was replaced by the rise of more restricted polymeric components.

These observations suggested that the most highly crosslinked gels take their elasticity not from entropically elastic dissolved chains, but from a crowded and jammed scaffold of collapsed material, as schematically illustrated in Figure 5b. The SEM results presented in the next section will confirm this assumption further.

### 3.5. Polymer Network Morphology Examined by SEM

**Figure 7:**
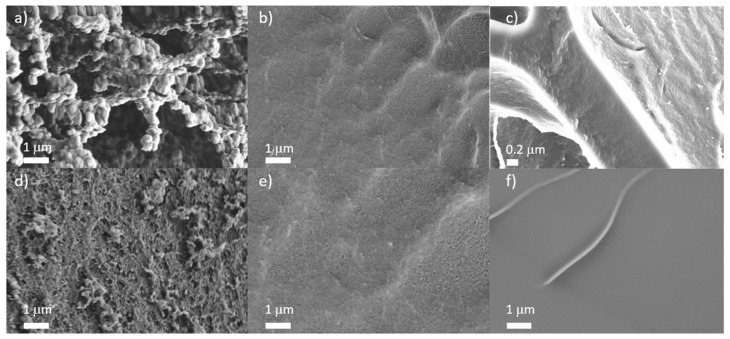
SEM images of freeze-dried hydrogels of poly(ECPA-*co*-BNEAA) (17/3) prepared in different solvents: (**a**) iPrOH; (**b**) PG; (**c**) EtOH/H_2_O (50/50), and freeze-dried hydrogels obtained from PG with different CL content: (**d**) poly(ECPA-*co*-BNEAA) (15/5); (**e**) poly(ECPA-*co*-BNEAA) (17/3); and (**f**) poly(ECPA-*co*-BNEAA) (19/1).

The dialyzed hydrogels were freeze-dried and the morphology was imaged by SEM to find out whether or not the pores indicated in Figure 5. could be visualized. SEM is able to detect pores larger than 10 nm. The pore size was determined by measuring in the SEM images. Figure 7a–c illustrate the morphology of networks with comparable chemical composition obtained from different solvents. The morphology strongly depended on the solvent. The most porous structures were obtained in networks from iPrOH (Figure 7a). This is caused by the fact that polymerization in iPrOH resulted in precipitation of the polymer. In contrast, gels from PG were the most homogeneous (Figure 7b). Gels prepared in EtOH/water (Figure 7c) showed an in-between morphology.

Figure 7d–f illustrates the influence of the CL content in gels obtained from PG indicating that the pore size was significantly enhanced by rising CL content, in contrast to the original expectation that rising CL content would reduce the pore size. Figure 5b already illustrated that the use of high CL contents resulted in particle gels consisting of highly crosslinked regions that are interrupted by large voids. These voids became smaller with decreasing CL content.

**Figure 8:**
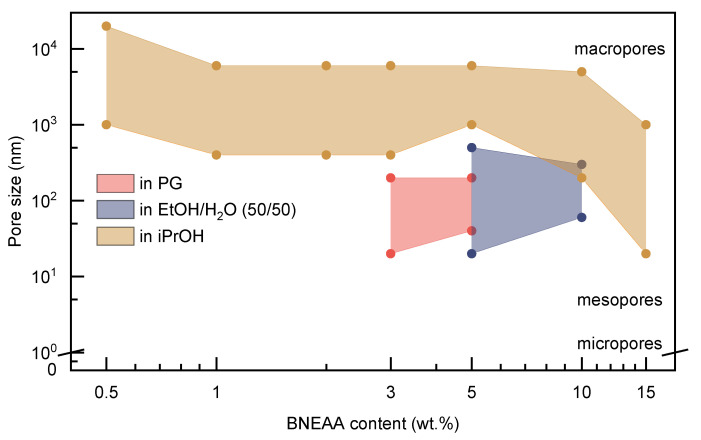
Typical pore sizes of freeze-dried poly(ECPA-*co*-BNEAA) hydrogels with varying composition and obtained from different solvents after dialysis.

Figure 8 summarizes the pore size ranges (lower line: minimum; upper line: maximum pore size found by SEM) of the gels with different CL content prepared in different solvents. Smaller CL contents yielded more distinct differences between the gels since the solubility of the poly(ECPA) homopolymer in the solvents is different (in iPrOH: not soluble; in PG: soluble; in EtOH/H_2_O (50/50): soluble). It was assumed that the behavior of gels with lower content of CL would be more similar to poly(ECPA) than to those with higher CL content.

To summarize, gels from iPrOH were characterized by the existence of mainly macropores, while gels from PG and EtOH/H_2_O showed meso- and macropores. Micropores could not be distinguished due to the resolution limit of SEM. The definition of micro-, meso- and macropores was applied according to IUPAC regulations regulations [36].

### 3.6. Buffering Behavior of the Polymer Networks

For later application in dentistry, a pH-buffering range ≥ 5.5 of the hydrogels is desired to achieve protection of tooth minerals from acid attacks. Therefore, the *pK_a_*_2_ of the hydrogels, which provides direct indication of the buffering behavior in this range, was determined. The *pK_a_* values of polyelectrolytes and polyelectrolyte networks can be distinguished into an external and internal *pK_a_* [37]. The external *pK_a_* is determined by the surrounding solution and will therefore be referred to as the “apparent” *pK_a,app_* in the further discussion. It can be determined by potentiometric titration. However, the external *pK_a, app_* values differ in close proximity of the polyelectrolyte chain, as known from Donnan theory [38], and becomes larger with increasing degree of crosslinking due to limited accessibility and slower diffusion rate of the ions [39,40]. The internal *pK_a_* can be calculated by Equation (4), which was established by Helferich et al. [37]:

(4) $$\bar{pK}=pH+\log\left(c\left(Na^{+}\right)\right)-\log\left(\frac{\bar{X}}{2}\right)$$

where $\bar{pK}$ is the internal *pK_a_*_2_ value of the polyelectrolyte gel, $\bar{X}$ is the concentration of ionizable groups of the polyelectrolyte gel and *Na^+^* is the sodium concentration at half-equivalence point.

**Figure 9:**
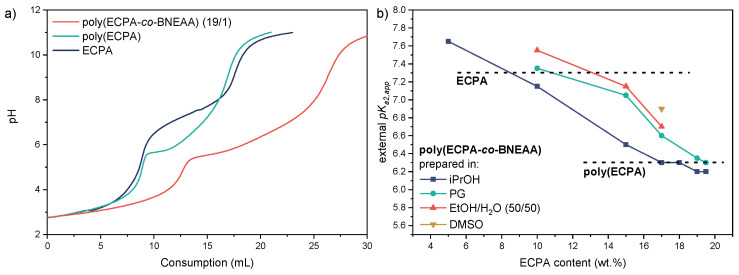
(**a**) Titration curves with 0.02 M Ca(OH)_2_ as titrant for ECPA (blue), poly(ECPA) (green) and the network poly(ECPA-*co*-BNEAA) (19/1) prepared in iPrOH (red), and (**b**) the external *pK_a_*_2*,app*_ values of the poly(ECPA-*co*-BNEAA) networks with various ECPA content prepared in different solvents.

Potentimetric titration with 0.02 M calcium hydroxide solution was employed to determine the external *pK_a_*_2*,app*_. Taking the restricted diffusion of dissolved ions in swollen networks into account, the time between the addition of portions of the titration agent was extended to 130 s. Ca(OH)_2_ as base was used since titrations with NaOH did not yield a second turnover point for the gel networks (see SM, Figure S3). Moreover, the use of Ca(OH)_2_ as base enables to simulate the conditions for subsequent dental applications (i.e., saliva). The use of NaOH presumably resulted in stretching of the polymer chains due to mutual repulsion by the homonymous charges [41,42]. The stretching of the polyelectrolyte molecules and the accompanying entropy reduction caused a lower protolysis tendency of the phosphonic acid groups and led to increased external *pK_a,app_* values compared to the corresponding monomer [22]. Figure 9a shows the comparison of titration curves and different external *pK_a_*_2*,app*_ of the monomer ECPA, the homopolymer poly(ECPA) and the selected network poly(ECPA-*co*-BNEAA)(19/1) prepared in iPrOH. The external *pK_a_*_2*,app*_ decreased from monomer to homopolymer to network, and the base consumption increased due to the restrictive diffusion of the solute ions.

The external *pK_a_*_2*,app*_ values of the gels should preferably be in the range ≤ 6.5 to obtain a buffer behavior in the pH range ≥ 5.5. This can be adjusted by increasing the crosslinker content and thus lowering the ECPA content in the network. Figure 9b shows the external *pK_a_*_2*,app*_ values of poly(ECPA-*co*-BNEAA) gels with different ECPA contents prepared in different solvents. Comparisons of theoretical and actual titrant consumptions to reach the turnover points suggested that all ionizable groups present in the gels were accessible and contributed to the buffering effect. This behavior was found for all gels studied, regardless of the CL concentration. According to Figure 9b, the external *pK_a_*_2*,app*_ values of the poly(ECPA-*co*-BNEAA) gels decreased with increasing ECPA content, and finally approached an external *pK_a_*_2*,app*_ value of about 6.3 which is comparable to that of the linear homopolymer poly(ECPA) (black dashed line in Figure 9b). That means, chain segments between crosslinks that are long enough behave as the linear homopolymer under the titration conditions used. At equal monomer/crosslinker ratios, gels prepared in iPrOH possessed the lowest external *pK_a_*_2*,app*_ value, followed by gels prepared in PG and EtOH/H_2_O (50/50). At a composition of 17/3 ECPA/BNEAA, gels prepared in DMSO showed the highest external *pK_a_*_2*,app*_ value. This confirmed the prediction of increasing external *pK_a_*_2*,app*_ values with increasing crosslinker content. By choosing appropriate ECPA/BNEAA ratios in a suitable solvent, gels can be prepared with external *pK_a_*_2*,app*_ values between 6.2 and 7.7 and pore sizes ≤ 10 nm to about 20 µm.

**Figure 10:**
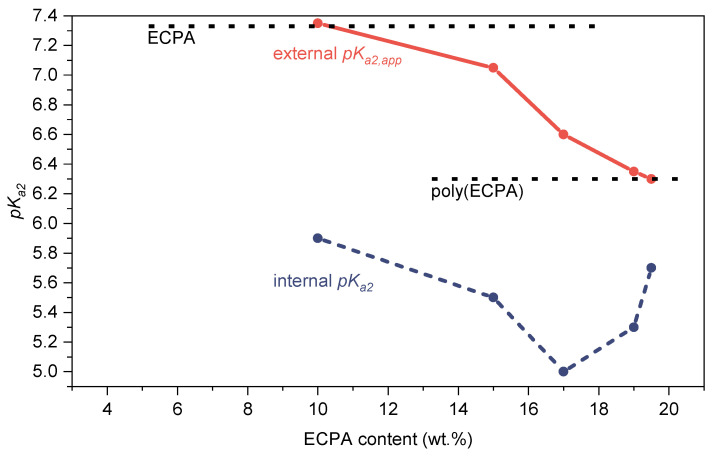
External *pK_a_*_2*,app*_ values (red) of the poly(ECPA-*co*-BNEAA) gels prepared in PG obtained by potentiometric titration compared with the internal *pK_a_*_2_ values (blue) calculated from Equation (4).

The internal *pK_a_*_2_ values of the poly(ECPA-*co*-BNEAA) gels prepared in PG were calculated using Equation (4) and transferring it to the system with calcium ions. Figure 10 compares the external *pK_a_*_2*,app*_ and calculated internal *pK_a_*_2_ values. The calculated internal *pK_a_*_2_ values inside the gels were clearly below the corresponding external *pK_a_*_2*,app*_ values, with differences ranging from 0.6 to 1.6 pH units depending on the CL content. The concentration of ionizable groups in gels which is decisive for the pH value inside the gels is influenced by both the swelling of the gel and the CL content. At a CL content of 3 wt.%, a minimum occurred in the calculated internal *pK_a_*_2_ curve, which was due to a comparatively high concentration of ECPA in the gel but low swelling. Below a CL content of 3 wt.%, the swelling of the gels increased, resulting in an increase of the internal *pK_a_*_2_ value.

The large differences between the obtained external *pK_a_*_2*,app*_ values and the calculated internal *pK_a_*_2_ values of the poly(ECPA-*co*-BNEAA) gels prepared in PG are problematic with regard to subsequent application in dentistry. Gels with a low crosslinker content and high degree of swelling would be most suitable, since a small difference between external and internal *pK_a_*_2_ occurs in the gel.

### 3.7. Determination of Calcium Enrichment in the Swollen Gels

The poly(ECPA-*co*-BNEAA) gels were studied for calcium enrichment, especially with respect to the formation of hydroxyapatite. Hydroxyapatite (Ca_5_(PO_4_)_3_OH) (HAP) is considered to be the main inorganic component of dentin [23,43]. Many studies report on the use of hydrogels as biomaterials for mineralization of various calcium compounds due to their biocompatibility, high water content and porous structure [43,44,45]. Dalas et al. [23] showed that phosphorus-containing polymers can enhance the HAP mineralization process. However, the mineralization process of HAP depends on the pH and is required to be above 6 and is therefore adjusted by buffer solutions in many studies [43,44,45,46]. Here, the poly(ECPA-*co*-BNEAA) networks showed buffering behaviour without additives and it was envisaged that the networks would take on this role.

**Figure 11:**
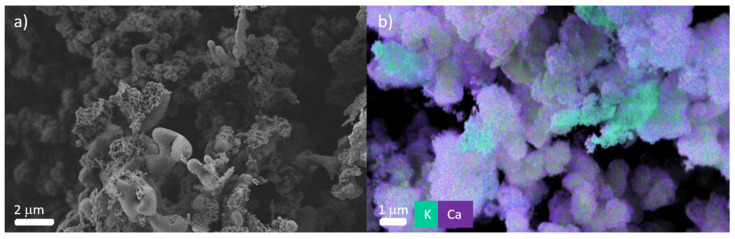
Images of poly(ECPA-*co*-BNEAA) (10/10) prepared in iPrOH after 24 h treatment in a double diffusion chamber recorded by (**a**) SEM and (**b**) EDX.

Poly(ECPA-*co*-BNEAA) gel films with different CL content prepared in iPrOH were placed in a double diffusion cell. The two separated chambers were filled with Ca(NO_3_)_2_ and K_3_PO_4_ solution, respectively (see SM, Figure S4). The gels were examined by SEM, EDX and WAXS for the deposition of calcium phosphate compounds. Figure 11 shows the SEM and EDX images of the poly(ECPA-*co*-BNEAA) (10/10) gel. In the SEM image (Figure 11a) crystalline structures could be observed throughout the cross-section of the gel sample, indicating the deposition of hydroxyapatite. Furthermore, EDX (Figure 11b) showed that potassium-rich phases also accumulated in the gel. Therefore, WAXS was carried out in order to determine the compounds present in the poly(ECPA-*co*-BNEAA) gels.

**Figure 12:**
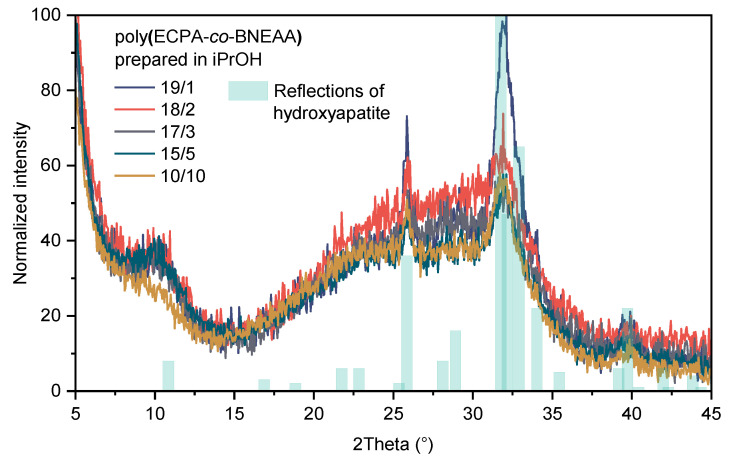
WAXS patterns of the poly(ECPA-*co*-BNEAA) gels of different compositions after 24 h treatment in the double diffusion chamber to examine the hydroxyapatite accumulation in the gel.

Figure 12 shows the WAXS patterns of the poly(ECPA-*co*-BNEAA) gels of different composition after exposure to Ca(NO_3_)_2_ and K_3_PO_4_ solutions in the double diffusion cell. The main reflections of hydroxyapatite according to the literature [47] (marked light blue in Figure 12) were observed in all gels. The intensity of hydroxyapatite increased with decreasing CL content. This correlates with the prediction that low CL content leads to a narrow difference between the internal and external pH of the gels, as discussed above, and thus, to preferential mineralization and accumulation of hydroxyapatite in the gel. Potassium-containing crystalline phases could not be detected in the WAXS patterns, indicating that the deposits containing potassium were either amorphous or too small crystallites.

## 4. Conclusions

Solvogel polymer networks (poly(ECPA-*co*-BNEAA)) based on the monomer ECPA and the crosslinker BNEAA were successfully synthesized by *in situ* UV-induced cross-linking polymerization in different solvents or solvent mixtures. Time-dependent *in situ* ATR-FTIR spectroscopy was used to analyze the polymerization process of the phosphonic monomer ECPA and demonstrated double bond conversion of the monomer which increased with increasing polarity of the solvent used for preparation. The influence of the content of crosslinker BNEAA as well as of the type of solvent on the formation of macroscopic gels was studied.

The morphology was characterized by *in situ* photorheology, solid-state ^1^H NMR spectroscopy and SEM. The gelation time and network parameters were obtained by *in situ* photorheology experiments. *G′* (i.e., the stiffness of the free-standing hydrogel samples) increased with increasing CL content and decreasing polarity of the solvent used for preparation. Solid state NMR spectroscopy revealed the existence of particle gels for networks with high CL content, while networks with CL of ≤1 wt.% BNEAA represented swollen yet rather inhomogeneous elastomers with a small fraction of actual elastically active polymer chains.

SEM showed that the solvent used for preparation had a significant influence of the morphology of dried poly(ECPA-*co*-BNEAA) gels. Solvogels prepared in iPrOH had predominantly macropores, while gels prepared in PG and EtOH/H_2_O were characterized by meso- and micropores.

The external and internal *pK_a_*_2_ values of poly(ECPA-*co*-BNEAA) networks were determined by a specially designed potentiometric titration. All gels showed buffering behavior. By choosing appropriate ECPA/BNEAA ratios in a suitable solvent, gels could be prepared with internal *pK_a_*_2_ values between 6.2 and 7.7 and pore sizes ≤ 10 nm to about 20 µm. The examined external *pK_a_*_2_ and the calculated internal *pK_a_*_2_ showed the smallest differences in gels with low CL content.

Calcium enrichment studies were carried out in a double diffusion cell and the resulting materials were characterized by SEM, EDX and WAXS. Hydroxyapatite mineralization could be observed in all gels with increasing intensity by decreasing CL content due to the presumed higher internal *pK_a_*_2_ in the gel.

The results obtained in the present study demonstrated that ECPA is a suitable monomer for the preparation of particle gels. The resulting hydrogels induced buffering behavior in a pH region interesting for dental applications. Under suitable conditions, hydroxyapatite can be mineralized on the hydrogel surface. Thus, the polymer networks can have potential applications as scaffold material for biomineralization and for the suppression of acid attacks on dentin material.

## Figures and Tables

**Table 1:** Conversion of double bonds of ECPA (20 wt.% in solvent, 0.5 wt.% initiator TPO) after different UV exposure times obtained by ATR-FTIR spectroscopy.

Solvent	PG	EtOH/H_2_O (50/50)	EtOH/H_2_O (80/20)	EtOH	iPrOH	DMSO
Monomer (mol%)	7.4	2.5	3.4	4.6	5.6	7.6
Double bond conversion (%) after *t*						
5 min	22	64	45	34	78	58
10 min	22	85	58	39	82	75
15 min	22	86	65	43	82	82

**Table 2:** Chemical composition of selected polymer networks compared to starting monomer compositions.

Ratio of ECPA/BNEAA in Solution (wt.%)	Solvent	Mass Loss in Dialysis (wt.%)	Components in Dialysate	Ratio of ECPA/BNEAA in Network (wt.%)
19.5/0.5	PG	5.3	PG	19.5/0.5
17.0/3.0	EtOH/H_2_O (50/50)	0.0	-	17.0/3.0
19.5/0.5	iPrOH	13.3	ECPA, poly(ECPA)	16.8/0.5
17.0/3.0	DMSO	0.0		17.0/3.0

**Table 3:** Gelation times of monomer/CL formulations with different ECPA/BNEAA ratios and in different solvents (monomer/solvent ratio: 20/80 wt.%/wt.%) obtained in photorheological measurements.

Ratio ECPA/BNEAA (wt.%/wt.%)						
Solvent	0/20	10/10	15/5	17/3	19/1	19.5/0.5
Gelation time (s)						
PG	^(a)^	6.8	9.3	10.0	12.7	19.9
EtOH/H_2_O (50/50)	^(a)^	13.2	26.2	25.6	^(a)^	^(a)^
EtOH/H_2_O (80/20)	4.7	16.6	31.5	^(a)^	^(a)^	^(a)^
iPrOH	^(a)^	10.2	^(b)^	^(b)^	^(a)^	^(a)^

^(a)^ Not examined; ^(b)^ gelation rheologically not detected (only visually).

## Data Availability

The data can be found at https://cloud.ipfdd.de/getlink/fiKoapCuQ4KH3vLowqjADYj1/Supplementary%20Material%20Heinze%20et%20al.pdf (accessed on 5 September 2021).

## Supplementary Materials

The following are available online at https://www.mdpi.com/article/10.3390/polym13203506/s1. Figure S1. ATR-FTIR cell for monitoring the UV polymerization in solution; Figure S2. Photorheological investigation of the gel formation in different sol-vents and with different crosslinker contents; Figure S3. Titration curves (**a**) of (**1**) poly(ECPA-*co*-BNEAA) (10/10), (**2**) poly(ECPA) and (**3**) ECPA with NaOH and (**b**) poly(ECPA) in NaOH and Ca(OH)_2_ for comparison with: (**1**) first titration with NaOH, (**2**) second titration with NaOH, (**3**) first titration with Ca(OH)_2_, (**4**) second titration with Ca(OH)_2_, (**5**) back titration with HCl after NaOH and (**6**) back titration with HCl after Ca(OH)_2_; Figure S4. Double diffusion cell in open state (left) and closed state (right); Table S1. Monomer Feed Compositions for the Synthesized poly(ECPA-*co*-BNEAA) gels with 80 wt.% solvent and 0.5 wt.% TPO as initiator based on the total mass; Table S2. Influence of monomer (ECPA)/crosslinker (BNEAA) ratio and solvent on the formation of macroscopic gels; Table S3. Storage moduli *G′* of the photorheologically investigated monomer mixtures or polyelectrolyte gels formed from them.
