# Supplementary material for: Polymer Networks for Enrichment of Calcium Ions
Source: Polymers (Basel). 2021 Oct 12;13(20):3506. doi: 10.3390/polym13203506 (PMC8538138; doi:10.3390/polym13203506)
Supplement: Supplementary file 1 [file polymers-13-03506-s001.zip › polymers-1390774-SI.pdf]

polymers

MDPI

Article

Polymer networks for enrichment of calcium ions

Marcus Heinze [1,2,3], Christoph Horn [1,2], Doris Pospiech [1,*], Regine Boldt [1], Oliver Kobsch [1], Kathrin Eckstein [1], Dieter Jehnichen [1], Brigitte Voit [1,2], Stefan Baudis [4], Robert Liska [4], Anna Naumova [5], Kay Saalwächter [5], Urs Lendenmann [6] and Norbert Moszner [6]

[1] Leibniz-Institut für Polymerforschung Dresden e.V., Hohe Str. 6, 01069 Dresden, Germany; marcus.heinze@better-basics.de (M.H.); horn-christoph@ipfdd.de (C.H.); pospiech@ipfdd.de (D.P.); boldt@ipfdd.de (R.H.); kobsch@ipfdd.de (O.K.); eckstein@ipfdd.de (K.E.); djeh@ipfdd.de (D.J.); voit@ipfdd.de (B.V.)
[2] Technische Universität Dresden, Organic Chemistry of Polymers, 01069 Dresden, Germany; horn-christoph@ipfdd.de (C.H.); voit@ipfdd.de (B.V.)
[3] Better Basics Laborbedarf GmbH, Zwickauer Str. 69, 01159 Dresden; marcus.heinze@better-basics.de (M.H.)
[4] Technische Universität Wien, Institut für Angewandte Synthesechemie, Getreidemarkt 9/163, 1060 Wien, Austria; stefan.baudis@tuwien.ac.at (S.B.); robert.liska@tuwien.ac.at (R.L.)
[5] Martin-Luther Universität Halle-Wittenberg, Institut für Physik, NMR, Betty-Heimann-Str. 7, 06120 Halle/Saale, Germany; anna.naumova@physik.uni-halle (A.N.); kay.saalwaechter@physik.uni-halle.de (K.S.)
[6] Ivoclar Vivadent AG, Bendererstr. 2, FL-9494 Schaan, FL (Urs.Lendenmann@ivoclarvivadent.com (U.L.); norbert.moszner@ivoclarvivadent.com (N.M.)
* Correspondence: pospiech@ipfdd.de; Tel.: +49-351-465-8497

Supplementary Material

Figure S1. ATR-FTIR cell for monitoring the UV polymerization in solution.

Figure S2. Photorheological investigation of the poly(ECPA-*co*-BNEAA) gel formation prepared in different solvents and with various crosslinker contents.

Figure S3. Titration curves (a) of (**1**) poly(ECPA-*co*-BNEAA) (10/10), (**2**) poly(ECPA) and (**3**) ECPA with NaOH and (b) poly(ECPA) in NaOH and $Ca(OH)_2$ for comparison with: (**1**) first titration with NaOH, (**2**) second titration with NaOH, (**3**) first titration with $Ca(OH)_2$, (**4**) second titration with $Ca(OH)_2$, (**5**) back titration with HCl after NaOH and (**6**) back titration with HCl after $Ca(OH)_2$.

Figure S4. Double diffusion cell in open state (left) and closed state (right).

Table S1. Monomer Feed Compositions for the Synthesized poly(ECPA-*co*-BNEAA) gels with 80 wt.% solvent and 0.5 wt.% TPO as initiator based on the total mass.

Table S2. Influence of monomer (ECPA)/crosslinker (BNEAA) ratio and solvent on the formation of macroscopic gels.

Table S3. Storage moduli *G′* of the photorheologically investigated monomer mixtures or polyelectrolyte gels formed from them.

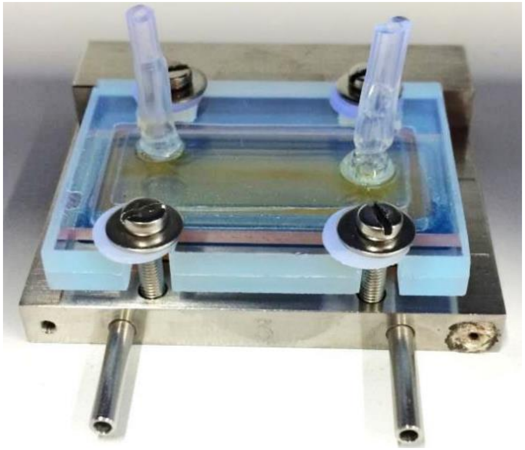

Figure S1. ATR-FTIR cell for monitoring the UV polymerization in solution.

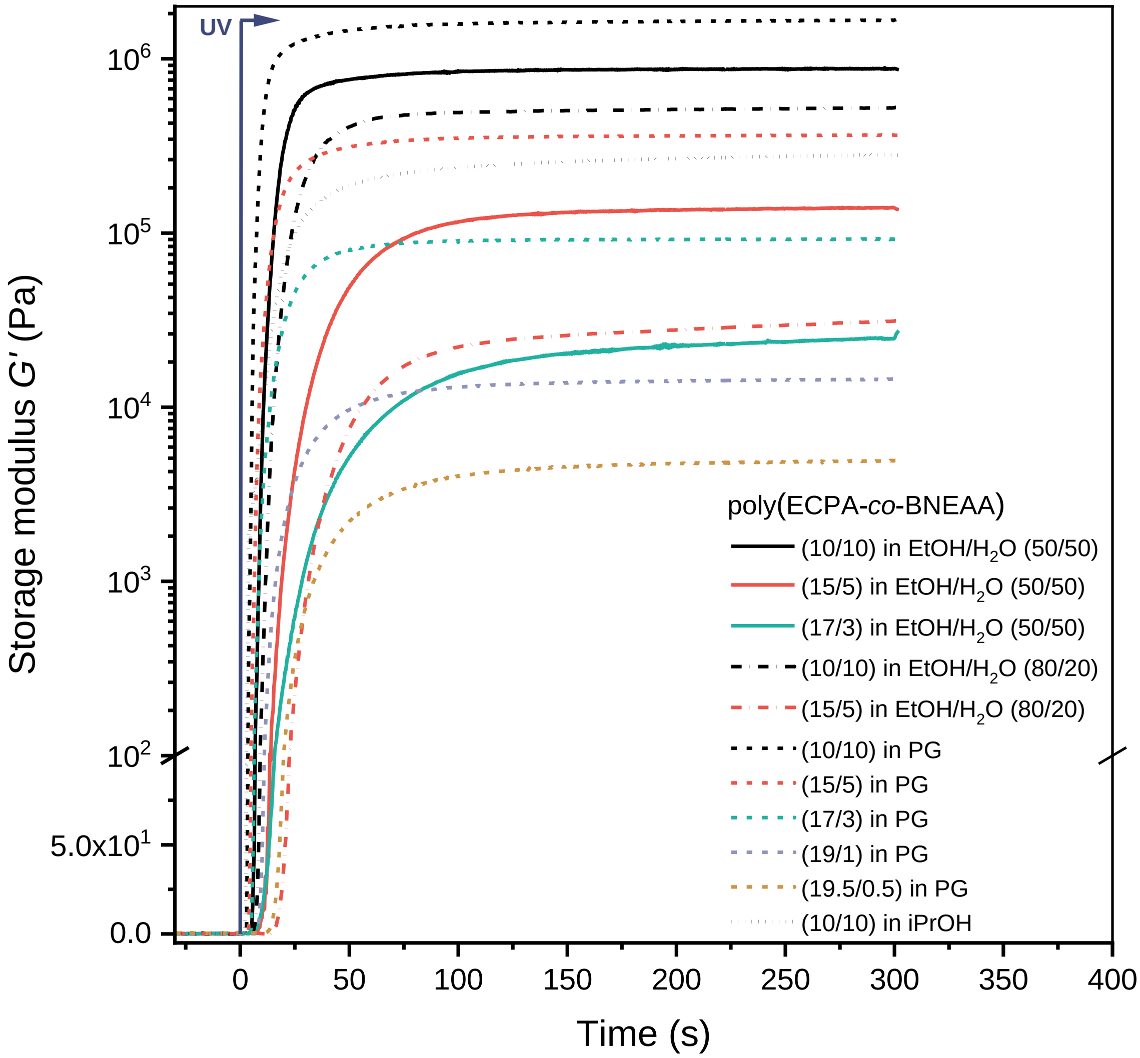

Figure S2. Photorheological investigation of the poly(ECPA-*co*-BNEAA) gel formation prepared in different solvents and with various crosslinker contents.

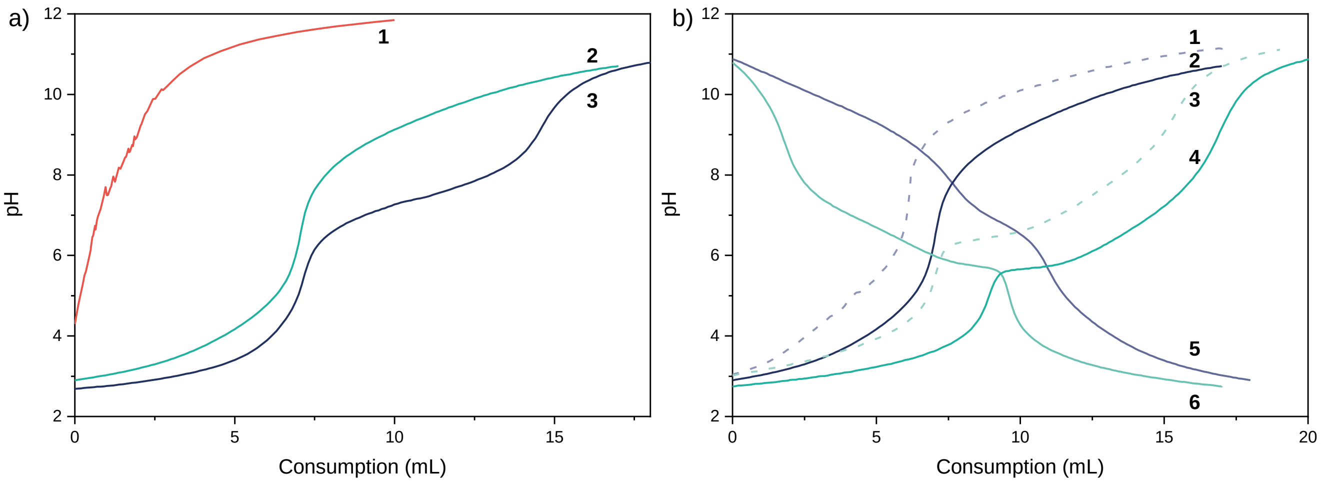

Figure S3. Titration curves (a) of (**1**) poly(ECPA-*co*-BNEAA) (10/10), (**2**) poly(ECPA) and (**3**) ECPA with NaOH and (b) poly(ECPA) in NaOH and $Ca(OH)_2$ for comparison with: (**1**) first titration with NaOH, (**2**) second titration with NaOH, (**3**) first titration with $Ca(OH)_2$, (**4**) second titration with $Ca(OH)_2$, (**5**) back titration with HCl after NaOH and (**6**) back titration with HCl after $Ca(OH)_2$.

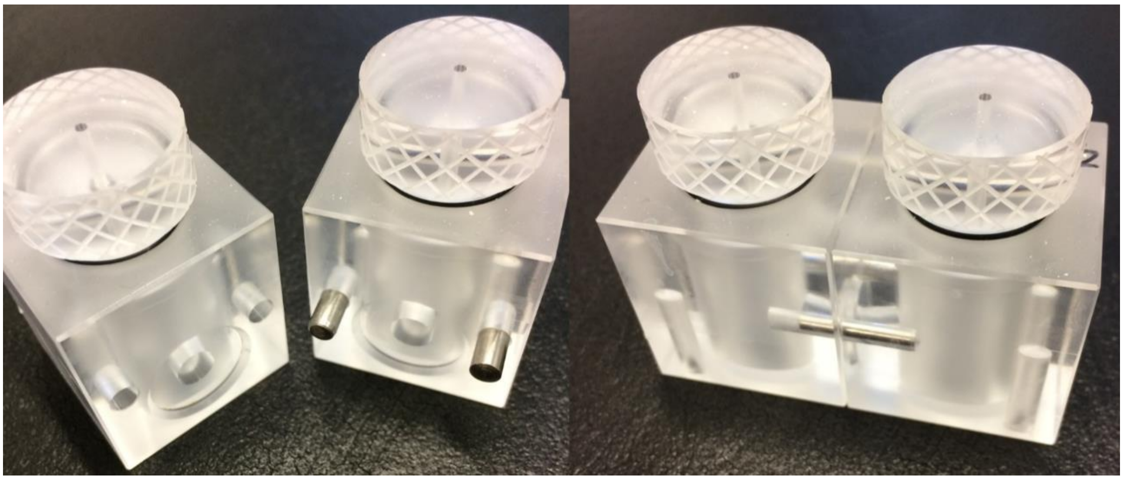

Figure S4. Double diffusion cell in open state (left) and closed state (right).

Table S1. Monomer Feed Compositions for the Synthesized poly(ECPA-*co*-BNEAA) gels with 80 wt.% solvent and 0.5 wt.% TPO as initiator based on the total mass.

Poly(ECPA-*co*-BNEAA)	ECPA (wt.%)	BNEAA (wt.%)
10/10	10	10
15/5	15	5
17/3	17	3
18/2	18	2
19/1	19	1
19.5/0.5	19.5	0.5

Table S2. Influence of monomer (ECPA)/crosslinker (BNEAA) ratio and solvent on the formation of macroscopic gels after UV irradiation for 60 s.

Ratio of ECPA/BNEAA in solution (wt.%)	Solvent			
	PG	EtOH/H_2O (50/50)	iPrOH	DMSO
10/10	Gel, firm	Gel, firm	Gel, firm	Gel, firm
15/5	Gel, firm	Gel, medium	Gel, firm	Gel, medium
16/4		Gel, soft		Gel, soft
17/3	Gel, firm	Gel, soft	Gel, soft	Gel, very soft
17/3	Gel	Gel, soft		Gel, soft
18/2	Gel	No gel		Gel, soft
19/1	Gel, medium	No gel	Gel, very soft	No gel
19.5/0.5	Gel, soft		Gel, very soft	
19.9/0.1	No gel		Gel, extremely soft	

Table S3. Storage moduli *G′* of the photorheologically investigated monomer mixtures or polyelectrolyte gels formed from them.

Solvent	Ratio of ECPA/BNEAA in solution (wt.%)					
	10/10	15/5	17/3	19/1	19.5/0.5	20/0
	Maximum Storage modulus *G′* (kPA)					
PG	1660	364	93	14	5	
EtOH/H_2O (50/50)	881	139	25			
EtOH/H_2O (80/20)	522	31				a)
iPrOH	282	a)	a)			

a) Gel formation not rheologically detectable.
